# Measuring epistemic success of a biodiversity citizen science program: A citation study

**DOI:** 10.1371/journal.pone.0258350

**Published:** 2021-10-11

**Authors:** Baptiste Bedessem, Romain Julliard, Eleonora Montuschi

**Affiliations:** 1 Dipartimento di Filosofia e beni culturali, Università Ca’Foscari, Venezia, Italy; 2 CESCO, Muséum National d’Histoire Naturelle, Paris, France; University of Granada: Universidad de Granada, SPAIN

## Abstract

This paper offers a comparative evaluation of the scientific impact of a citizen science program in ecology, ‘‘Vigie-Nature”, managed by the French National Museum of Natural History. Vigie-Nature consists of a national network of amateur observatories dedicated to a participative study of biodiversity in France that has been running for the last twenty years. We collected 123 articles published by Vigie-Nature in international peer-reviewed journals between 2007 and 2019, and computed the yearly amount of citations of these articles between 0–12 years post-publication. We then compared this body of citations with the number of yearly citations relative to the ensemble of the articles published in ecology and indexed in the ‘‘Web of Science” data-base. Using a longitudinal data analysis, we could observe that the yearly number of citations of the Vigie-Nature articles is significantly higher than that of the other publications in the same domain. Furthermore, this excess of citations tends to steadily grow over time: Vigie-Nature publications are about 1.5 times more cited 3 years after publication, and 3 times more cited 11 years post-publication. These results suggest that large-scale biodiversity citizen science projects are susceptible to reach a high epistemic impact, when managed in specific ways which need to be clarified through further investigations.

## Introduction

The participation of non-professional scientists in the production of scientific knowledge is hardly a new phenomenon. The history of the natural sciences, from XIX^th^ century botany to contemporary ecology and conservation sciences, shows several instances of amators engagement in scientific practices ([1, 2]). However, calls for a larger implication of lay citizens in the process of knowledge and expertise production are on the rise, while inclusiveness in scientific research is increasingly valued by institutional discourse [3]. Within scientific practices themselves, this trend is particularly notable through the growth of so-called ‘‘citizen science”, which includes a large variety of forms of participation of non professional scientists (citizens, some members of NGOs) in the production of scientific knowledge [4].

The value of this type of public engagement in science for citizen empowerment [5], public learning and understanding of science ([6, 7]), and the development of new tools of governance [5, 8, 9] is well recognized. However, the growth of citizen science practices goes hand in hand with epistemic concerns about whether and how ‘‘the public can make actual contributions to science” [10]. For instance, doubts have been raised about the quality of the data collected in crowdsourcing programs [11, 12], or about the neutrality of the participants which sometimes pursue a political agenda [13]. In this context, a fundamental question for most scientists concerns the ability of citizen science projects to deliver results that can significantly advance scientific knowledge [14–18]. In response to these concerns, many contributions have been recently proposed, which aim to demonstrate the epistemic value of citizen science. This is notably the case in ecology and environmental science, where participative practices have proved their utility for biodiversity evaluation [19], the knowledge and management of natural resources [20, 21], or the assessment of ecosystemic services [21]. Given these purported successes, a more general question emerges about the conditions which maximize the scientific ‘‘impact and reuse” of citizen science data and results [22]. In this context, the systematic evaluation of the scientific outputs of citizen science projects is a central challenge in terms of how both to identify good participative practices and to increase the trust of the whole scientific community in the ability of those projects to produce reliable and useful knowledge. However, if the epistemic evaluation of research is a complex and controversial issue in general [23], it seems to be even trickier in the case of citizen science. Among the reasons put forward by [24]: the results produced by citizen science projects are not always presented and disseminated in the form of peer-reviewed scientific publications, which despite limitations, are normally adopted as ‘‘raw material” for scientometric studies aiming to evaluate scientific productivity [15]. A further reason that is advocated for is the technical difficulty of gathering large numbers of existing scientific publications produced by citizen science projects, since these are rarely labeled as such in literature database such as Web Of Science or Google Scholar [25].

Recently, [15] collected 143 publications in peer-reviewed journals resulting from 23 distinct citizen science projects in the field of astronomy. He conducted a citation study of these articles over a 15-year period, and found that their citation rate achieves a peak within three years from publication, by which time they have been significantly more cited than the average astronomy papers. This result is interpreted in terms of scientific ‘‘impact” both by the author and by a data-brief published in *Nature Index* [26]: citizen science publications in astronomy have a strong short-term relative impact, and this impact decreases on the mid and long-term. Of course, the very notion of scientific impact is an abstract construct which can endorse different meanings, and be quantified through different types of measurements, for instance by using social network analysis [27]. However, the citation rate (the number of citations of a given article in subsequently published papers) is an old tool [28] which is still largely used as a proxy for the impact of scientific production, even if it is well recognized that it constitutes a partial and imperfect variable for research evaluation [29], including ecological science [30].

In this paper, we aim to contribute to the debate about the evaluation of the epistemic value of public engagement in scientific research by conducting a citation study of citizen science-based publications in the field of ecology and conservation science. Following [15], we consider the temporal evolution of the citation rate as a proxy for the scientific impact of a given publication. However, while [15] considered an aggregate of heterogeneous citizen science projects, our study draws on one large-scale, long-term citizen science program: the Vigie-Nature network of participative observatories of biodiversity, managed since 1989 by the Muséum National d’Histoire Naturelle (MNHN) in Paris, France.

## Materials and methods

### The ‘‘Vigie-Nature” program

Vigie-Nature started in 1989, with the aim of improving our knowledge of biodiversity by engaging a large diversity of people (amateur naturalists, managers of green areas, pupils, farmers and other citizens) in the collection of field data in the whole French territory (for more information, one can refer to http://www.vigienature.fr/fr/presentation-2831). Vigie-Nature mobilizes non-professional citizens to quantify the abundance of species, in order to characterize the evolution of ecosystems in terms of species assemblage. It gathers different participative observatories, focusing notably on pollinator insects (*Spipoll* project), birds (*BirdLab*), or urban vegetation (*Sauvage de ma rue*).

The program is managed by the MNHN, with the collaboration of naturalist associations. Data quality is controlled by scientific experts, which verify species’ identifications. The researchers use these collected data to advance research in ecology and conservation science by publishing results in scientific journals such as *Biodiversity and Conservation*, *Journal of Ornithology*, *Biological Conservation*, *Landscape Ecology*, *Global Change Biology*, and *Frontiers in Ecology and the Environment*. Between 2007 and 2019, 171 articles were published within the frame of this program. The corresponding references are available in a dedicated data base (https://www.zotero.org/groups/85788/vigienature/items/collectionKey/4M3I4ZUR). Within this body of publications, 123 articles present scientific results on the basis of collected data, while the remaining articles propose meta-reflections about citizen science. The Vigie-Nature program is thus well adapted for the purpose of scientometric studies since it allows to pursue statistical analysis on the basis of a large sample of publications. Whereas studies such as Odenwald’s (2018) rely on aggregated data from distinct citizen science programs, the bulk of Vigie-Nature’s publications is large enough to allow for a study of the scientific impact of one specific project.

### Data collection

**Fig 1:**
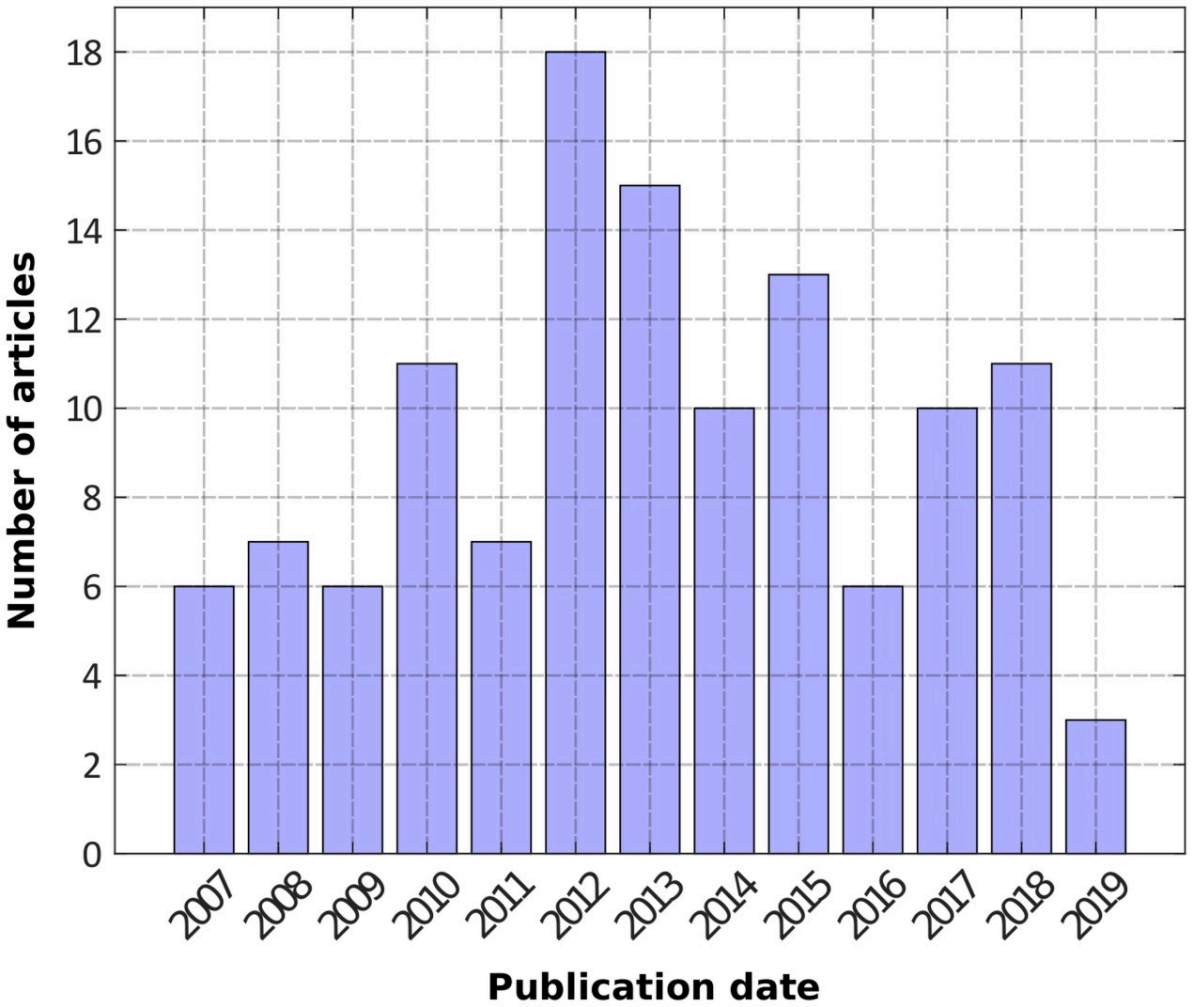
Number of Vigie-Nature articles per year of publication. *NB*: At the time of our analysis, the whole data for 2019 were not yet available, which explains the relative low number of articles for this year.

To conduct our study, we considered the 123 scientific articles in the field of ecology and environmental sciences from the Vigie-Nature bibliography published between 2007 and 2019, with a total of 5813 citations in peer-reviewed journals (hereafter, VN group of articles). Fig 1 gives the distribution of this sample with respect to publication year.

To collect the number of citations on each year post-publication for each of these publications we used the Web Of Science (WoS) database—a reliable and user friendly platform, widely considered the ‘‘gold standard” for citation studies [31]. We first computed the number of self-citations (we considered an article A is self-cited by B when at least one author of A is also an author of B) in order to allow for a more comprehensive quantification of the scientific impact of Vigie-Nature’s result. We found a self-citation rate of about 11% in average, which appears to be close to the average value of self-citations in contemporary science (about 12% following [32]), but lower than the self-citation rate in the ecological sciences (about 16% following [33]). S2 Appendix compares the citation profile obtained with and without self-citation. In the following, we do not exclude self-citation from the citation rates.

We then calculated the number of citations for each article from their year of publication (shifted to year 0) up to 2019. This produces data on a range from 0 to 12 years post-publication, with 123 articles for the first point (time = 0 year) and 6 articles (corresponding to those published in 2007) for the last one (time = 12 years). This method of calculation might introduce a bias because a paper published, for instance, on 2019-01-01 is treated as identical to a paper published on 2020-12-31. This bias may be expected to be influential for the first point of the yearly citation profile (time = 0 years). However, we suggest that it rapidly attenuates after this point. Besides, we suggest that this effect operates similarly for the Vigie-Nature samples and for the comparison groups of papers. S1 Appendix presents simulation results which strengthen the relevance of this approximation.

This set of data was compared with the citation history of three bibliographic ensembles. The first one consists of the articles published between 2007 and 2019 and referenced in WoS in the category ‘‘Biodiversity Conservation” (sample size: 60847 articles, hereafter BC group of articles). This sample was constituted by using the filters ‘‘Biodiversity Conservation” (as a WoS category), ‘‘2007–2019”, and ‘‘Article” (as document type).

For every publication year, the annual citation rate of these articles were then extracted by creating a ‘‘citation report” in WoS. This citation report contains, for a given year, the total number of articles under the BC category, and the yearly number of citations from 2007 to 2019. These data are given in S3 Appendix. We then computed an average citation rate per year post-publication following the same methods as the one used for Vigie-Nature (VN) articles.

The second set of data includes all the articles published between 2007 and 2019 within the ensemble of scientific journals where the Vigie-Nature articles are published (hereafter, VN journals groups). To build Fig 3, the citation history of every VN article was compared to the average citation history of the articles published in the corresponding journal. More precisely, for each article *A* from the Vigie-Nature bibliography, published in the journal *J* during the year Y, and which got, *t* years after publication, a number of citations N_A_(t), we computed R = N_A_(t)/N_j_(t), with N_j_(t) being the average number of citations *t* years after publication of the articles in the journal *J* during the same year Y. This ratio R represents, for each VN article, its relative amount of citations compared with the whole publications in the same journal. Journals’ citation histories are easily extracted from WoS by creating a ‘‘citation report” (after selecting the publication year, the document type, and the journal title). Let us note that some journals are not referenced in WoS, and were not taken into account in the statistical analysis (namely: *Diversity*; *Current Zoology*; *Nature Climate Change*; *Peer-Review Journal*; *Computational management science*; *Methods in ecology and evolution*; *Urban ecosystems*; *Regional Environment change*; *British birds*; *Ecology and evolution*; *Conservation Letters*; *Insect Conservation and Diversity*). 17 Vigie-Nature articles are then excluded. Besides, we also excluded from the analysis articles published in generalist journals, namely: *Science*, *Nature*, *Proceeding of the Royal Society*: *Biology*, *Biology Letters*, *Plos One*. 13 VN articles were then removed from the analysis. Finally, this second data set includes 78988 articles. Third, we also used the articles from the ‘‘Environment and Ecology” category from Clarivate Analytics. Between 2009 and 2019, this category gathers 841 673 articles. We directly extracted some statistical figures from the data base (citation thresholds for the 10%, 20% and 50% most cited papers. These data are available here: https://esi.clarivate.com/BaselineAction.action).

#### Statistical analysis

All the calculations, and in particular statistical tests were carried out by using Matlab 2019b. The statistics were developed through a longitudinal analysis using the *mvregress* function in Matlab (specifically conceived to lead this kind of longitudinal analysis aiming to compare temporal series by modeling them under the form of polynomials). With this function, we operated multivariate regressions to fit, with different models, the time-series of yearly citations for the different groups of papers: VN (Vigie-Nature articles), BC (Biology and conservation articles), and VN journals articles. In its more complex form, the regression model takes into account the time *T* (0 to 12 years), the origin *O* of the articles (BC, VN or VN journals), and the interaction between time and origin *T*x*O*. For a two-degree polynomial, the global equation model is the following:

$${\text{y}}_{\text{ij}}=\beta_{\text{o}}+\beta_{1}{\text{G}}_{\text{i}}+\beta_{2}{\text{t}}_{\text{ij}}+\beta_{3}{\text{t}}_{\text{ij}}{}^{2}+\beta_{4}{\text{G}}_{\text{i}}{\text{t}}_{\text{ij}}+\beta_{5}{\text{G}}_{\text{i}}{\text{t}}_{\text{ij}}{}^{2}+\varepsilon_{\text{ij}}$$

with y_ij_ the value (number of citations) for article *i* at time t_ij_ (in years), G_i_ the origin of the paper (equal to 1 for VN and 0 for BC or VN journals), ε_ij_ the error in fitting y_ij_, and (β_o_.…β_5_) the coefficients to be found. Apart from this global model, two reduced models can be designed: the first one ignores the *O* variable (MR1), and the other one ignores only the interaction terms β_4_ and β_5_ (MR2). The *mvregress* function then finds the β terms and the likelihood objective function for these models (the global one and the reduced ones). We then led two kinds of analysis. First, for the general comparison of citation history, we computed the MR1 model and we compared it to the global model which includes *O* as a variable. We then compared these two models in their ability to fit the data, by using a likelihood ratio test (using the *chi2cdf* function in Matlab). The null hypothesis is that the two models are equivalent, which would mean that the reduced model without *O* is sufficient, and thus that the origin of the paper does not determine the citation history.

Second, for the analysis of the evolution of the relative citation rate of Vigie-Nature articles, we compared MR2 and the global model (which includes interaction terms). We applied the same likelihood ratio test to determine if the interaction of time and origine had a significant effect on the citation history profile. In other terms, this analysis allows to determine if the differences measured in the yearly citation rate change in time. The results we obtained are presented in the next section.

## Results

### Comparison of citation history

**Fig 2:**
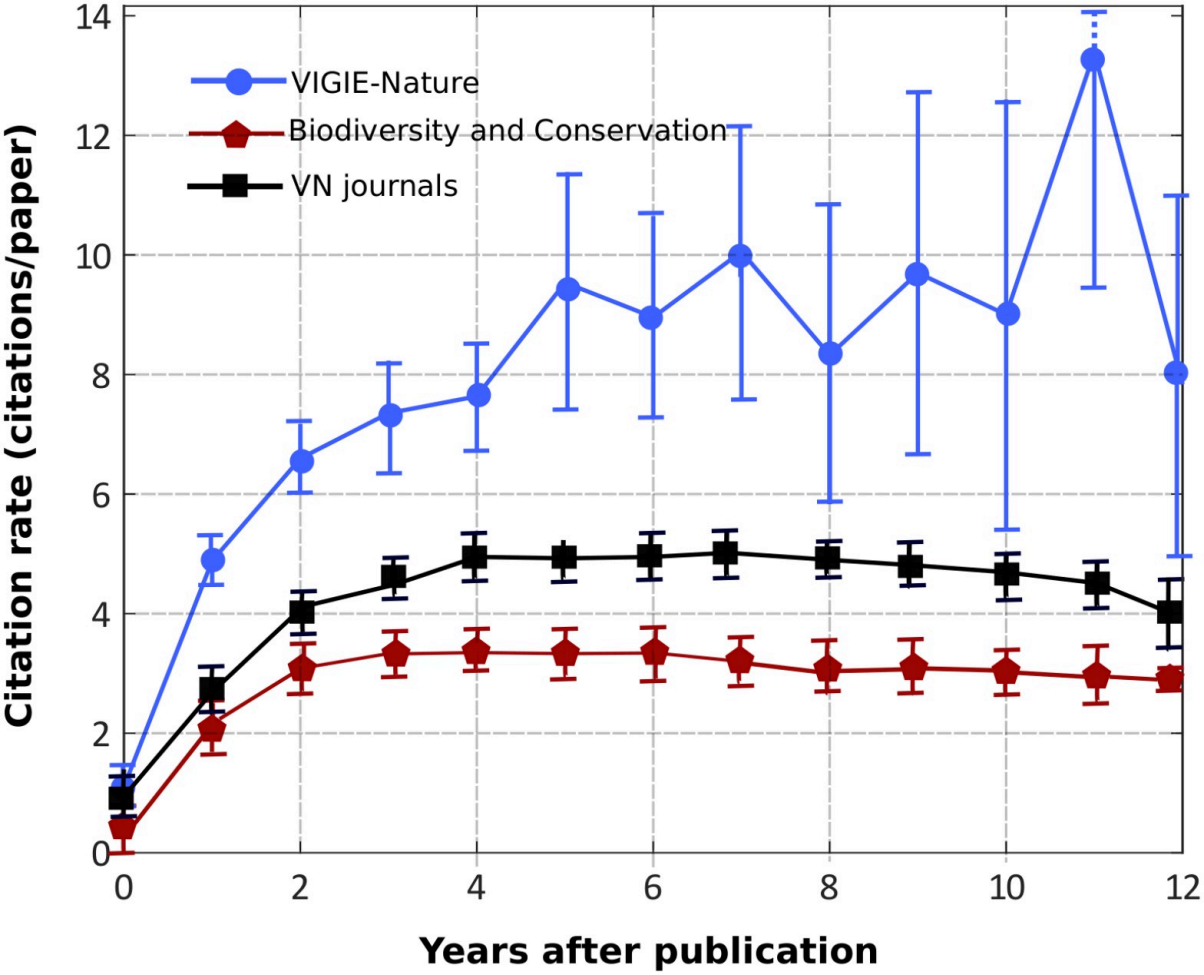
Comparative citation rate history. We plotted the average citation history for Vigie-Nature’s articles (blue, total sample size: 123 articles), for the ‘Biodiversity and Conservation’ articles from WoS (red, sample size: 60847 articles), and for the articles from the Vigie-Nature journals group (black, sample size: 78988 articles).

We first computed the citation history for the 123 papers extracted from the Vigie-Nature (VN) bibliographic database. We calculated the average number of annual citations as a function of the time post-publication from year 0 to year 12. We present the corresponding graph on Fig 2. Error bars are classically calculated as σ/(N^0.5), with σ the standard deviation and N the sample size. In Table 1, we give the number of values used to calculate each point (depending on the number of available articles, see Fig 1).

**Table 1:** Sample size corresponding to the Vigie-Nature curve in Fig 1 (points from 0 to 12 years).

	0	1	2	3	4	5	6	7	8	9	10	11	12
**N**	123	120	109	99	93	80	70	55	37	30	19	13	6

On the same figure we drew the average annual amount of citations for the articles in the ‘‘Biodiversity Conservation” (BC) category of WoS. We also drew the citation history for the articles from the VN journal group. The average citation history profile of these two groups of standard (non citizen science based) research articles shows an increase in the amount of annual citations during the first 4 years after publications to reach a plateau of about 3 citations/year from year 4 up to year 12 post-publication. The average citation history of Vigie-Nature articles seems to follow a similar profile, but with an ‘‘excess” of citations from 0 to 12 years post-publication. To test the significance of this effect, we led a longitudinal analysis of the citation history (see the ‘‘Method” section for details). Using a multivariate regression (to fit the data with a polynomial function using a maximum likelihood estimation), we first tested if the VN and BC profiles are significantly distinct. To do so, we compared two models in their ability to fit the data (see the Methods section for details). This test gives a p-value of 0.005. We thus accept the hypothesis that taking into account the origin of the paper improves the fit: in other words, we conclude that the difference between the Vigie-Nature citation history and the Biodiversity and conservation one is significant with a risk inferior to 5%. We performed the same test to compare VN and VN journals articles. We found a p-value<10^−5^, which shows that the Vigie-Nature citation history is also significantly distinct from the VN journals one.

In addition, it seems that this difference in the time-series of yearly citations grows with time. In the next sub-section we explore the hypothesis that the relative long-term impact of Vigie-Nature articles (compared to standard ones) is higher than their relative short-term impact.

### Evolution of the relative amount of citations of Vigie-Nature articles

**Fig 3:**
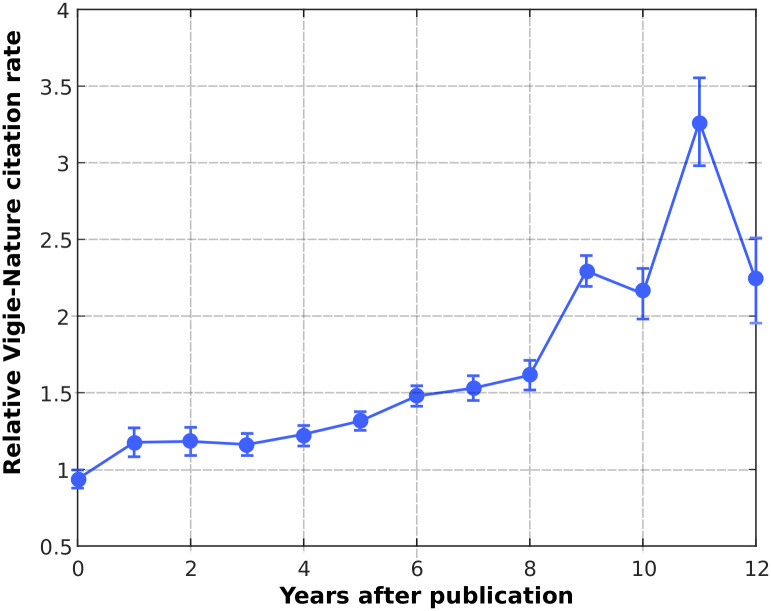
Average relative yearly citation rate of the Vigie-Nature articles. We plotted the relative amount of annual citation of Vigie-Nature articles with respect to the ensemble of articles published in the same journals during the same year.

In order to visualize this time-dependant ‘‘excess” of citations, we computed the average relative amount (R) of Vigie-Nature annual citations, as explained in the Methods section: for each VN article, the number of citation is divided by the average annual citations of the papers published in the corresponding journal, as a function of time (Fig 3). The results indicate that the average relative amount of citations for VN articles seems to be superior to 1 from 0 to 12 years, which is coherent with our previous analysis. Furthermore, it seems that the ‘‘excess” of citations of VN articles grows with time, at least between 0 and 11 years. Indeed, the ratio after 11 years is significantly higher that the ratio between 0 and 4 years (3.3 against about 1.4). This might indicate that the differences between Vigie-Nature articles and ‘‘mainstream” ones in terms of impact become more relevant in the long-term. To test the significance of this effect, we extend the longitudinal analysis presented in the previous section (see the ‘‘Method” section for full details). As for the comparison of citation history, we compared two models in their ability to fit the data. The first one (model 1) does not consider the interaction terms between time *T* and origin *O* of the papers, and the second one introduces interaction terms. We then led a likelihood-ratio test in order to test the null hypothesis that the two models are equivalent. For the comparison between the BC and VN groups, the test gives a p-values of 0.026 (<0.05). We thus accept the hypothesis that taking into account the interactions between *T* and *O* improves the fit. This means that the VN and BC citation history profiles present different tendencies (that is to say, curves in Fig 2 are not similar, in the sense that they are not ‘‘parallel”). The same test performed for the VN and VN journals groups gives a p-value<10^−6^, which confirms the previous results. By comparing this result to the evolution of the average relative VN citation rates (Fig 3), it seems reasonable to argue that these difference in general tendencies are due to a time-dependant growing ‘‘excess” of citation of Vigie-Nature articles. In other words, the relative impact of VN articles (compared to ‘‘mainstream” ones published in the same journals) might significantly grows with time (at least from 0 to 11 years).

### Statistical analyses of the dispersion of the citation rates

**Fig 4:**
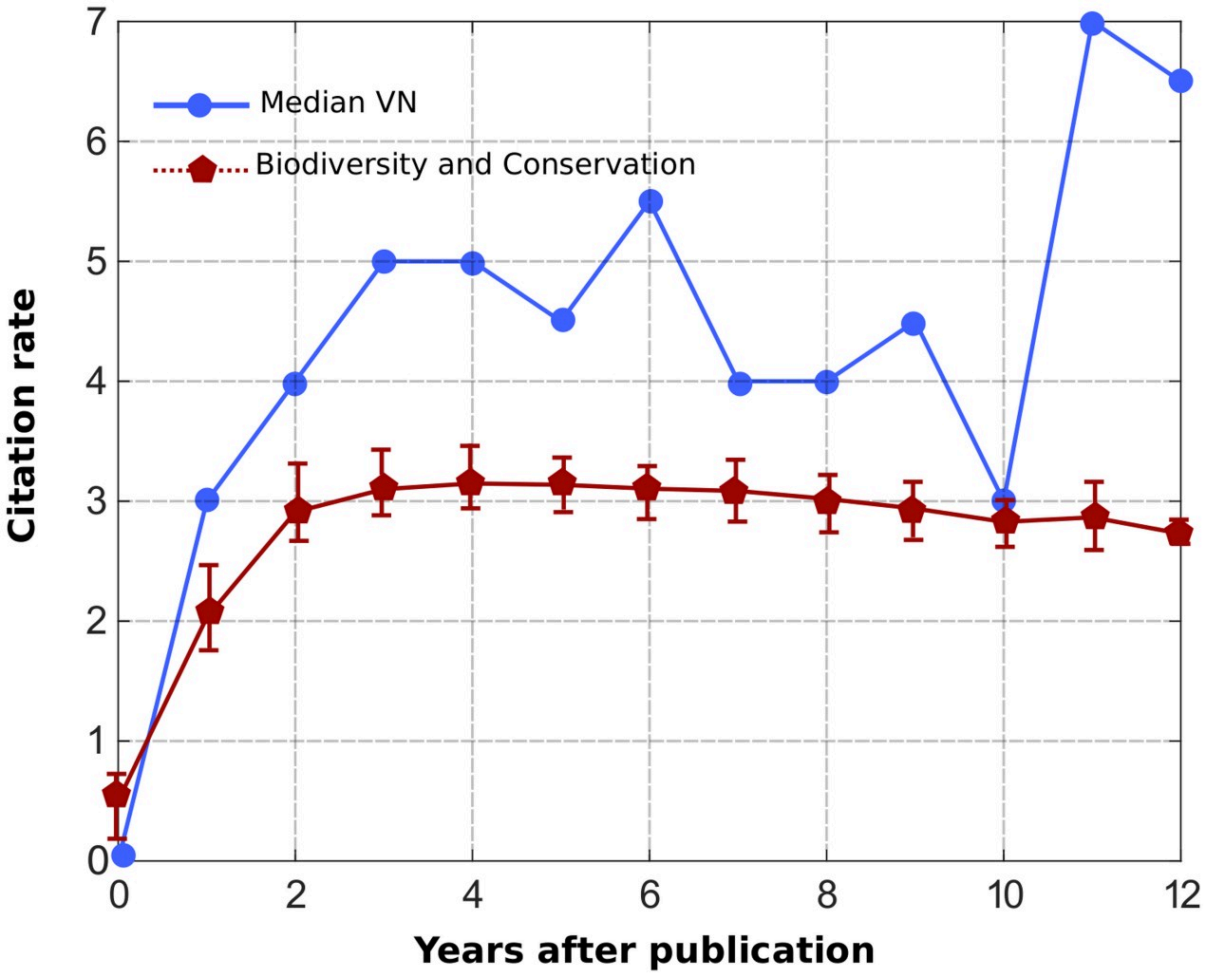
Time-dependent median value of the Vigie-Nature articles citation rate. The median (in blue) is compared to the average citation rate of the articles in biodiversity and conservation from WoS (in red, sample size: 60847 papers).

At this point, an issue can be raised about the statistical distribution of the overall ‘‘excess” of citations of Vigie-Nature articles. Does it only concern a few highly-cited articles, or is it a more equally distributed feature regarding Vigie-Nature publications? To get a more precise picture of the citation profile, we computed the value of the median of the set of Vigie-Nature articles citations at each time *t* post-publication (that is to say, the number *X* of citations such as half of the papers are more cited than *X*, *t* years after publication). It appears that from 0 to 12 years, the median value is slightly higher than the average citation rate of the articles in the ‘‘Biodiversity and Conservation” category from WoS (see Fig 4). This means that about half of the Vigie-Nature articles are annually more cited than the mainstream publications in the same area. We also computed the deciles partition of the total number of citations of Vigie-Nature articles. Table 2 presents the citation thresholds of the 10%, 20% and 50% most cited papers both for Vigie-Nature and the whole ‘‘Environment and Ecology” category (from Clarivate Analytics) between 2009 and 2019. The 9^th^ decile (the 10% threshold) is about 2.3 times higher in the Vigie-Nature set of articles, whereas the 5^th^ decile (the 50% threshold) is about 3.5 times higher. Together, these results suggest that the ‘‘excess” of citations of Vigie-Nature productions is quite equally distributed among the articles. At the very least, the average citation history of Vigie-Nature papers is clearly not led by a small amount of highly cited ones.

**Table 2:** Citation thresholds for the 10%, 20% and 50% most cited papers, for Vigie-Nature and the whole Environment and Ecology field, between 2009 and 2019.

	10%	20%	50%
Environment/Ecology	33	19	6
Vigie-Nature	76	50	21

## Interpretation and discussion

We have computed the citation history of 123 papers published in peer-review journals (between 2007 and 2019) resulting from Vigie-Nature, a long-term and large-scale citizen science program in ecology and environment sciences. We found that Vigie-Nature articles are on average significantly more cited from 0 to 12 years after publication than the total of articles in the same domain. This average value refers not only to a few highly cited papers. In addition, the difference in yearly citations rate strongly increases with time. We evaluated that between 0 and 4 years post-publication, Vigie-Nature publications are cited each year about 1.4 times more than the mainstream ones (BC and VN journals groups). After 11 years, they are on average cited 3.3 times more per year. Quite interestingly, this long-term impact strongly contrasts with what was obtained by [15] when studying the citation history of publications issued from citizen science projects in astronomy. The author shows that the aggregated citation rate of 143 published papers resulting from 23 citizen science projects is very high 3 years after publication (about 4 times the average of a sample of classical papers). This short-term peak is followed by a sharp decline: after 8 years, citizen science articles from his sample are significantly less cited than mainstream ones. However, our results are fare for being directly comparable to [15]’s ones, since the projects’ structures and scientific domain are very different.

At this stage, a central issue is that of the generality of the conclusions we can draw from our results for citizen science practices. Vigie-Nature is indeed a very specific program, characterized by its large scale (the whole French territory) and its longevity (more than thirty years). In addition, the produced data are massively diffused through peer-reviewed scientific articles, which is rarely the case for biodiversity citizen science projects: [34] have shown that only 12% of the 388 projects they surveyed reach scientific literature. In addition, they suggest that the public availability of the data, the control of scientific rigor, and the projects’ spatial scale and longevity positively influence publication. Similarly, [35] have shown that the accessibility of the data, and the presence of coordinators aiming to maintain data quality enhance the scientific impact of biodiversity citizen science projects. Furthermore, projects dedicated to long-term monitoring also seem to have a greater impact. Together with these studies, our results might allow to formulate some hypothesis about the conditions which promote the epistemic success of biodiversity citizen science projects. First, projects exhibiting a relatively large temporal and spatial extent appear to have a (relatively) high scientific impact. Here, it would be relevant to compare the impact of Vigie-Nature with that of *non-participative* large-scale, long-term networks of observatories of biodiversity, such as the countryside survey ([36], https://countrysidesurvey.org.uk/) in UK or the worldwide CTFS-forestgeo ([37], https://forestgeo.si.edu/) project. Second, the management structure of the projects might also be a central determinant of their epistemic success. Vigie-Nature is characterized by a very centralized structure, with a few number of well-identified researchers managing a vast network of observatories and a vast amount of collected data. These managers are assisted locally by naturalist associations which recruit participants and animate the programs by organizing meetings and by sending newsletters. This structure might enhance the scientific impact of the projects because data are collected, controlled and used by a stable, dedicated team of researchers. These ones got a good expertise in producing peer-reviewed papers on the basis of participative data collection. Apart from this specific structure of the citizen science programs, the constitution of the research team might also play a role in itself, notably through its professional quality, its notoriety, its insertion within the research networks, etc. To have a more complete view of the factors influencing epistemic success of biodiversity citizen science project, it would then be necessary to address a range of comparisons with other similar participative projects in ecology—such as, for instance, the eBird program ([38], https://ebird.org/home) from Cornell Laboratory of Ornithology.

We also should keep in mind that scientific impact cannot be the only determinant of what should count as ‘‘good practices” of participation. The ethical issue of the visibility of the participants should also be considered seriously. As noted by [39], various citizen science programs in environmental sciences still make contributing citizens invisible in scientific productions, in spite of advances in securing data quality. More generally, this lack of recognition of volunteer contributors is a well-recognized ethical problem [40, 41]. Yet, Vigie-Nature productions do not explicitly include recognition of citizen contributions. In addition, this lack of recognition also questions the influence of citizens’ invisibility on the diffusion of the corresponding scientific results [39]. As many scientists manifest a lack of trust as respects to citizen science data and results [42], the fact that participants are made invisible in Vigie-Nature productions might contribute to their epistemic impact. This would be a crucial point to consider in the future of this citizen science program. Some inspiring propositions have been made in literature regarding the online sharing of biodiversity data. For instance, [43] insist on the importance of recognizing the work of data publishers when citing datasets in scientific papers. This suggestion points to an explicit reference to Vigie-Nature participatory datasets within the Vigie-Nature scientific publications. Moreover, Vigie-Nature data could be shared online through international biodiversity data networks, such as the Global Biodiversity Information Facility [44]. This would give a visibility to this specific type of data, and could increase the epistemic trust placed by researchers in citizen science.

## Supporting information

S1 Fig Comparison of the original sample citation profile (red curve) and the computed one (blue curve). The blue curve was obtained by applying the approximation method we use in our study. The sample size is *n* = 100 papers.
(TIF)

S2 Fig Evaluation of the robustness of our citation rate calculation. Average relative error generated by our method of calculation of the yearly citation rate as a function of the sample size.
(TIF)

S3 Fig Average yearly citation rate for the Vigie-Nature articles. The blue curve includes self-citations, and the red one excludes self-citations.
(TIF)

S1 Appendix Robustness of the calendar year method. (DOCX)

S2 Appendix Autocitation rate. (DOCX)

S3 Appendix (7Z)

S1 Data (RAR)
