# Supplementary figures and images for: Measuring epistemic success of a biodiversity citizen science program: A citation study
Source: PLoS One. 2021 Oct 11;16(10):e0258350. doi: 10.1371/journal.pone.0258350 (PMC8504750; doi:10.1371/journal.pone.0258350)

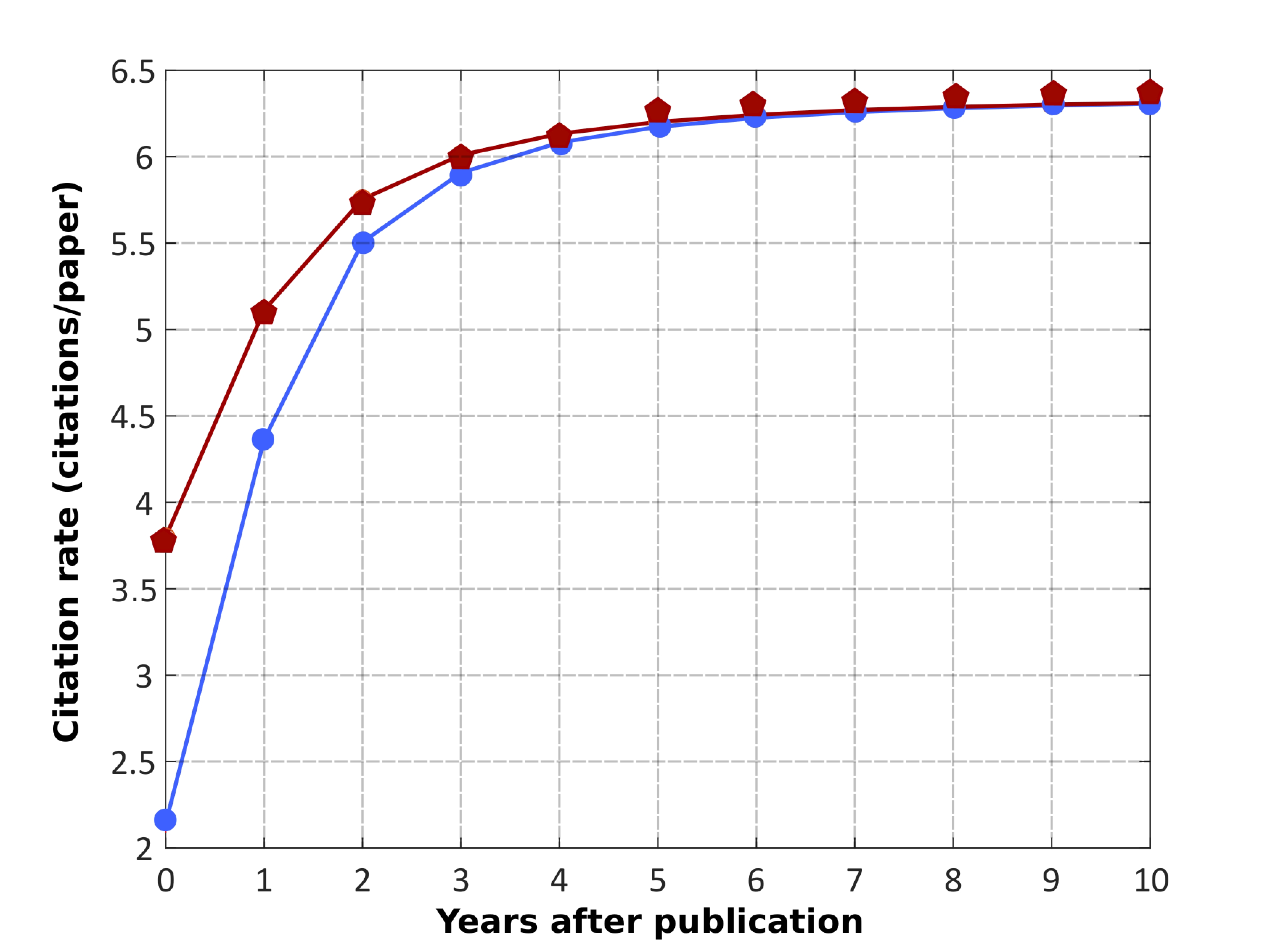

Supplement: S1 Fig — The blue curve was obtained by applying the approximation method we use in our study. The sample size is n = 100 papers. (TIF) [file pone.0258350.s001.tif]

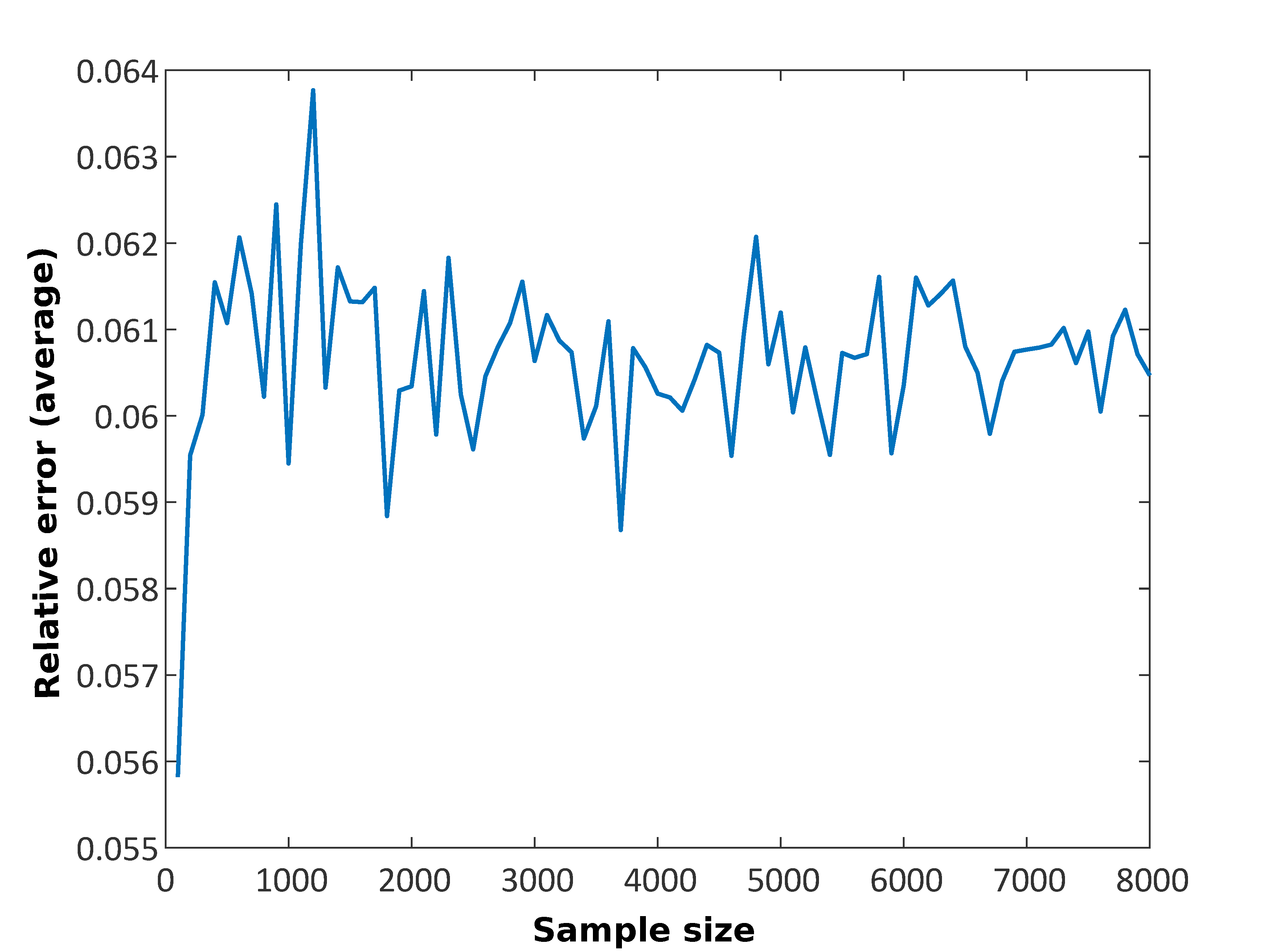

Supplement: S2 Fig — Average relative error generated by our method of calculation of the yearly citation rate as a function of the sample size. (TIF) [file pone.0258350.s002.tif]

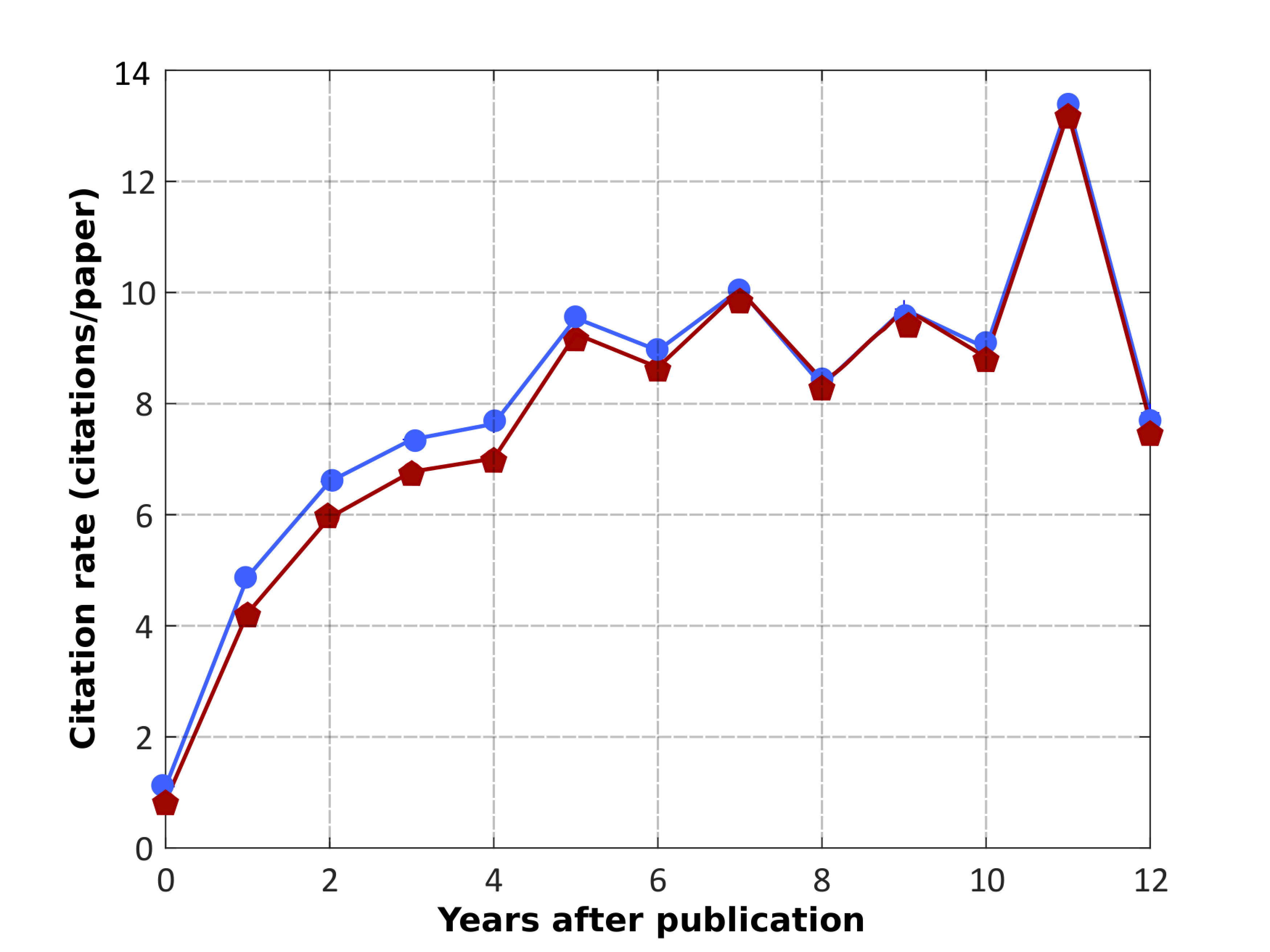

Supplement: S3 Fig — The blue curve includes self-citations, and the red one excludes self-citations. (TIF) [file pone.0258350.s003.tif]
