# Supplementary material for: Measuring epistemic success of a biodiversity citizen science program: A citation study
Source: PLoS One. 2021 Oct 11;16(10):e0258350. doi: 10.1371/journal.pone.0258350 (PMC8504750; doi:10.1371/journal.pone.0258350)
Supplement: S1 Appendix — (DOCX) [file pone.0258350.s004.docx]

**Appendix A Robustness of the calendar year method**

To assess the robustness of the method we used to calculate yearly citation rates, we created an artificial test sample of *n* articles published in a same given year. The months of publication were randomly chosen within the calendar year. The citation profiles were generated following equations y=c+a*x^2^/(x^2^+b) to reproduce the typical citation profile of ‘Environment and Ecology’ papers in Web of Science. The c,a,b parameters were randomly determined in ranges which were chosen to represent the variability of the citations profiles within this ‘Environment and Ecology’ category. We then applied to this sample the same method we use in our study: a paper published in a given month M1 of a year Y is considered identical as a paper published in a month M2 of the same year Y. We then compared the average profile obtained with this approximation to the original average profile. Fig 5 presents these two profiles, obtained for *n*=100 papers. As expected, our method underestimates the citation rate for the first point (year=0). However, this error becomes rapidly unsignificant.

We then tested the effect of the size *n* of the sample on the value of the gap between the original and the computed profiles. Fig 6 gives the average value of the relative gap between the two profiles (computed on the range [0 11 years]), as a function of the size *n* of the sample (computed on the range [100 8000]). The average presents a very narrow range of variation (around 6%). This result suggests that the error which is introduced by using our approximation method is restricted to the very first point (year=0), and that it weakly depends on the number of articles. Consequently, it may be supposed to be the same for the Vigie-Nature sample (*n*=123) and for the ‘Biodiversity and Conservation’ one (*n*=60857).
