# Supplementary material for: Measuring epistemic success of a biodiversity citizen science program: A citation study
Source: PLoS One. 2021 Oct 11;16(10):e0258350. doi: 10.1371/journal.pone.0258350 (PMC8504750; doi:10.1371/journal.pone.0258350)
Supplement: S2 Appendix — (DOCX) [file pone.0258350.s005.docx]

**Appendix B Autocitation rate**

We computed the yearly citation rate of the Vigie-Nature papers, with and without self-citation. Fig 7 compares the average profiles obtained for the 123 papers in these two cases.
